# Impact of ambient temperature on life loss per death from cardiovascular diseases: a multicenter study in central China

**DOI:** 10.1007/s11356-021-16888-7

**Published:** 2021-10-11

**Authors:** Ling-Shuang Lv, Chun-Liang Zhou, Dong-Hui Jin, Wen-Jun Ma, Tao Liu, Yi-Jun Xie, Yi-Qing Xu, Xing-E Zhang

**Affiliations:** 1 Hunan Provincial Center for Disease Control and Prevention, Changsha, 410005 China; 2 Guangdong Provincial Institute of Public Health, Guangdong Provincial Center for Disease Control and Prevention, Guangzhou, 511430 China; 3 Hunan Provincial Climate Center, Changsha, 410007 China

**Keywords:** Cardiovascular diseases, Ambient temperature, Extreme temperature, Year of life lost, Mortality, Distributed lag nonlinear model

## Abstract

**Background:**

In the context of global climate change, studies have focused on the ambient temperature and mortality of cardiovascular diseases (CVDs). However, little is known about the effect of ambient temperature on year of life lost (YLL), especially the life loss per death caused by ambient temperature. In this study, we aimed to assess the relationship between ambient temperature and life loss and estimate the impact of ambient temperature on life loss per death.

**Methods:**

We collected daily time series of mortality and meteorological data from 70 locations in Hunan province, central China, in periods ranging from Jan. 1, 2013, to Dec. 31, 2017. Crude rates of YLL were calculated per 100,000 people per year (YLL/100,000 population) for each location. A distributed lag nonlinear model and multivariate meta-regression were used to estimate the associations between ambient temperature and YLL rates. Then, the average life loss per death attributable to ambient temperature was calculated.

**Results:**

There were 711,484 CVD deaths recorded within the study period. The exposure-response curve between ambient temperature and YLL rates was inverted J or U-shaped. Relative to the minimum YLL rate temperature, the life loss risk of extreme cold temperature lasted for 10 to 12 days, whereas the risk of extreme hot temperature appeared immediately and lasted for 3 days. On average, the life loss per death attributable to non-optimum ambient temperatures was 1.89 (95% CI, 1.21-2.56) years. Life loss was mainly caused by cold temperature (1.13, 95% CI, 0.89‑1.37), particularly moderate cold (1.00, 95% CI, 0.78‑1.23). For demographic characteristics, the mean life loss per death was relatively higher for males (2.07, 95% CI, 1.44‑2.68) and younger populations (3.72, 95% CI, 2.06‑5.46) than for females (1.88, 95% CI, 1.21-2.57) and elderly people (1.69, 95% CI, 1.28-2.10), respectively.

**Conclusions:**

We found that both cold and hot temperatures significantly aggravated premature death from CVDs. Our results indicated that the whole range of effects of ambient temperature on CVDs should be given attention.

**Supplementary Information:**

The online version contains supplementary material available at 10.1007/s11356-021-16888-7.

## Introduction

Cardiovascular diseases (CVDs) are the leading cause of premature death globally. The Global Burden of Disease Study in 2019 estimated that a total of 9.6 million people died of CVDs, accounting for almost a third of all deaths globally (Collaborators GDaI 2020). The prevalence of CVDs in China has been persistently increasing and remains the major cause of death. In 2017, CVD-related deaths accounted for 45.91% and 43.56% of all deaths in rural and urban areas, respectively. Two out of five cases of death were attributed to CVDs (China NCfCD 2020).

Under the background of climate change, many studies have showed that climate change poses a catastrophic risk to human health and that heat waves result in excess mortality, usually seen in the exacerbation of CVDs (Cai et al. 2021; Yang et al. 2021). Ambient temperature is regarded as an important risk factor for public health (Gasparrini et al. 2015; Son et al. 2019). Recently, the majority of epidemiological studies have demonstrated association between non-optimum ambient temperature exposure and mortality or morbidity of CVDs (Achebak et al. 2019; Dimitrova et al. 2021; Huber et al. 2020; Martínez-Solanas and Basagaña 2019). The findings indicated that high or low temperatures could increase the risks of mortality or morbidity. One systematic review reported that the risk of cardiovascular mortality increased by 5% for cold exposure and 1.3% for heat exposure (Moghadamnia et al. 2017). However, those studies have focused on using death counts, relative risk, or attributable fraction as the primary outcome, which may not provide an ideal representation of the total mortality burden attributable to non-optimum temperatures. A major drawback of those health outcomes is that the measures give same the weight to every death occurring at very different ages.

One approach to avoid the shortcoming is to consider years of life lost (YLL) as the health outcome. YLL is a more informative indicator because it combines the number of deaths with age at death, and can give more weight to deaths among younger people (Huang et al. 2012). Compared with relative risk or attributable fraction, YLL can be combined with the number of deaths to calculate the mean life loss per death, which may directly measure the reduction in lifespan (Majdan et al. 2017). Some studies have estimated the exposure-response associations between ambient temperature and YLL (Egondi et al. 2015; Odhiambo Sewe et al. 2018). Recently, one national study reported life loss of CVDs per death attributable to ambient temperature, which used crude YLL rate (YLL per 100,000 population) as a novel measure. The results presented that both high and low temperatures could increase YLL rates of CVDs (Hu et al. 2021). However, the study cannot support more precise impact for specific region, especially for Hunan province, central China. Hunan province have complex and changeable climate, and temperature extremes exhibit a warming trend during the period of 1960‑2013 (Chen et al. 2018b). CVDs are the most serious causes of death in the province. So, the impact of ambient temperature on life loss per death from CVDs needs to be explored.

In this study, we conducted a time series analysis including 70 locations in Hunan province, central China. We aimed to explore the impact of ambient temperature on YLL rates, and calculate the mean life loss per death of CVDs attributable to non-optimum ambient temperature.

## Methods

### Study location and data collection

**Fig. 1:**
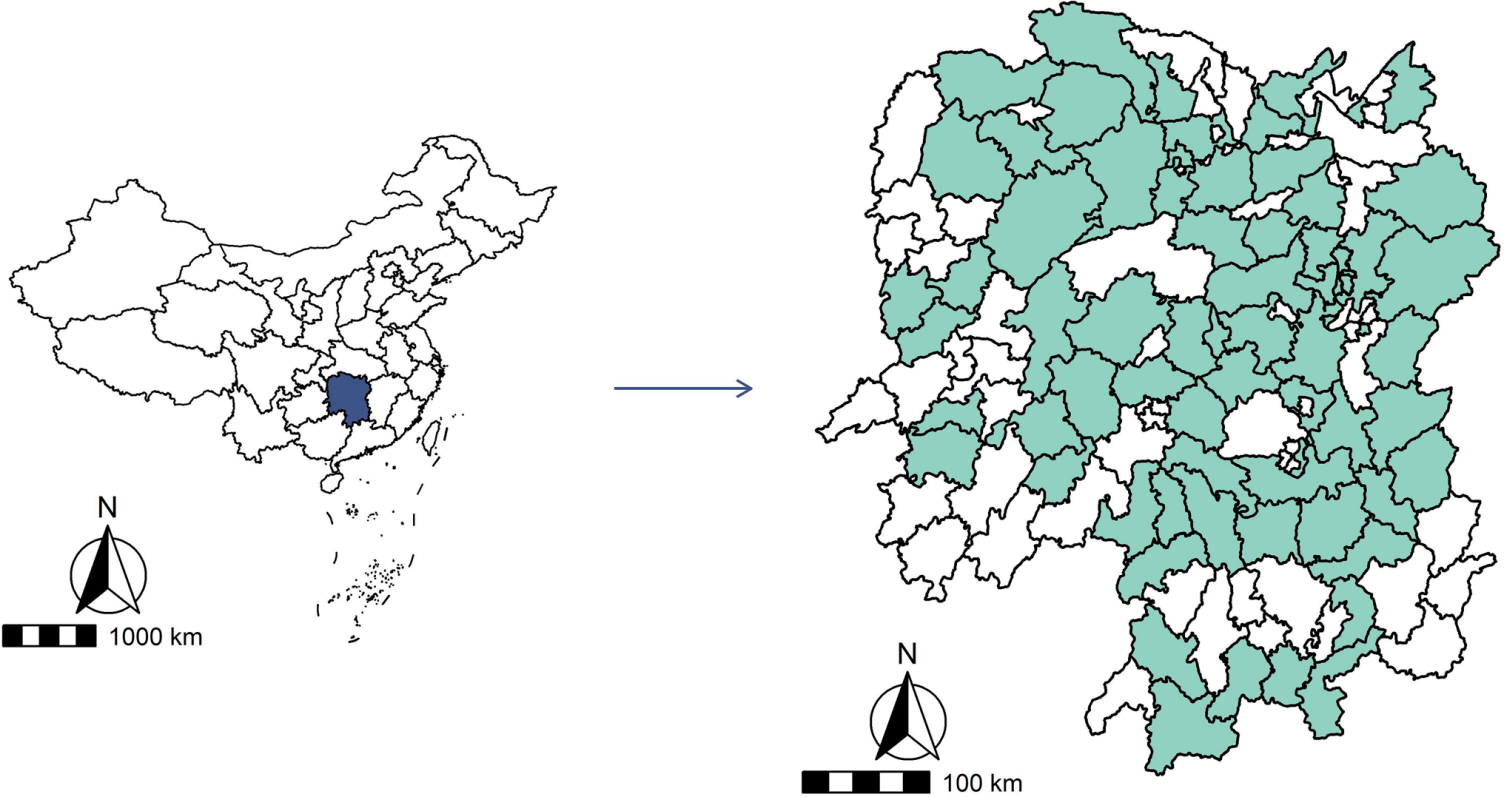
The geographical distribution of the 70 study sites in Hunan

Hunan province is located in central China at 24° 38′- 30° 08′ N, 108° 47′- 114° 15′ E, with an area of 211,800 km^2^. Our study included 70 locations (counties or districts) across Hunan province (Fig. 1). To ensure enough statistical power, only locations with a population size > 200,000 and/or an annual mortality rate > 4‰ were included (Li et al. 2021).

Daily cardiovascular disease mortality data from January 1, 2013, to December 31, 2017, were obtained. All deaths were classified into groups according to the International Classification of Diseases, 10th revision (ICD-10): total cardiovascular disease (CVD, I00–I99), hypertension (HP, I10‑I15), ischemic heart disease (IHD, I20‑I25), cerebrovascular disease (CED, I60‑I69), hemorrhagic stroke (HS, I60‑I62), and ischemic stroke (IS, I63). Information on the sex, date of birth, date of death, and cause of death were included.

Daily average ambient temperature and relative humidity from 698 meteorological monitoring stations across China were extracted from the China Meteorological Data Sharing Service System (http://data.cma.cn/). We used Australian National University Splines (Hutchinson and Xu 2013) to interpolate the daily mean temperature, minimum temperature, maximum temperature, and relative humidity at a 0.01° × 0.01° spatial resolution across China. We extracted the data of the grids where each selected location was covered and then calculated the average of grids for each location.

### YLL rates calculation

We obtained population size from the sixth national population census conducted in 2010 (China NBoSo 2012). Then, we computed the individual YLL by matching the age and sex of each death to the 100-year life table. The life table was presented in a previous article (Lv et al. 2020). For each location, daily total YLLs of CVD were calculated by summing all individual YLLs on the same day. After that, we stratified the daily YLLs by sex (male and female), age (0–64 and ≥ 65 years), and specific cause of death (HP, IHD, CED, HS, and IS). To adjust the effect of population size on total YLL in different locations, we used the YLL rate as a health outcome. Crude rates of YLL were calculated per 100,000 people for each location using the annual population (Luo et al. 2021; Majdan et al. 2017).

### Statistical analysis

We performed all analyses with the R software (Team RC 2020), using the packages *dlnm*, *mvmeta*, and *ggplot2*. Two-tailed *P* values less than 0.05 were considered statistically significant.

#### Estimation of the association between temperature and YLL rate

We used a two-stage approach to estimate the association between daily average ambient temperature and the YLL rate of CVDs.

In the first stage, a distributed lag nonlinear model (Gasparrini 2011) linked with a Gaussian distribution function was employed to investigate the location-specific effect, with adjustments for seasonality, long-term trends, day of the week, and daily humidity. Briefly, a natural cubic B-spline of time with 7 degrees of freedom (*dfs*) per year to control for seasonal and long-term trends, and a categorical variable to control the day of the week. We also employed a natural cubic B-spline with 3 *dfs* to adjust the potential confounding effect of relative humidity*.* A cross-basis function was introduced to model the complex nonlinear and lagged dependencies of the associations between ambient temperature and the YLL rate. Specifically, we modeled the nonlinear associations between ambient temperature and the YLL rate with a quadratic B-spline with three internal knots placed at the 10th, 50th, and 90th percentiles of city-specific daily mean ambient temperature distribution. We modeled the delayed effect with a natural cubic B-spline. We used 21 days as the maximum lag period for ambient temperature (Chen et al. 2018a; Gasparrini 2014).

In the second stage, we used a multivariate meta-analytical model (Gasparrini et al. 2012) to pool the best linear unbiased prediction of the location-specific overall cumulative exposure-response associations. The minimum YLL rate temperature (MYT), which corresponds to a minimum YLL rate percentile, was treated as the centring value of the association. We divided daily mean temperatures into four components, extreme cold (≤ 2.5th percentile), moderate cold (the 2.5th percentile to the MYT), moderate heat (MYT to the 97.5th percentile), and extreme heat (> 97.5th percentile). These cut-offs are consistent with previous studies (Chen et al. 2018a; Xu et al. 2019).

#### Calculation of the average life loss per death attributable to temperatures

We calculated the daily average YLL per CVD death caused by ambient temperature and its components based on the temperature-YLL rate relationship and population size of each location. The total YLLs attributable to ambient temperature were obtained by accumulating daily attributable YLLs. The average YLLs per death resulting from non-optimum temperature were calculated by dividing the total attributable YLLs by the number in the respective group or subgroup (Majdan et al. 2017). In order to explore the effect modification, *Z* test was performed to explore the difference between effect estimates for different subgroups. The ratio *z* = *d*/SE(*d*). *d* refers the difference of effect estimates for two categories. SE(*d*) refers the square root of the sum of two squares of the separate standard errors (Altman and Bland 2003; Yang et al. 2019).

#### Sensitivity analysis

A series of sensitivity analyses were conducted to check the consistency of the results. We employed maximum lag periods of 14, 21, and 28 days, changed the *dfs* of time from 6 to 8 per year, and explored the exposure-response relationship with or without PM_10_.

## Results

### General characteristics

The distribution of average daily YLL rates of CVDs, related subgroups and weather conditions in the selected cities within the study period are given in Table 1. In total, 711,484 CVDs deaths were recorded. The mean daily YLL rates for CVD-, CED-, HP-, HS-, IHD-, and IS-related mortality were 10.1, 3.7, 2.9, 1.6, 1.2, and 0.8 per 100,000 population per day, respectively. The average daily YLL rates for males was higher than those for females, and the elderly population (age 65+ years) had much higher YLL rates than the younger population (age 0–64 years). We observed large variations in daily ambient temperature with a mean of 17.7 °C, ranging from −3.5 to 34.5 °C. The mean relative humidity was 77.8%. Daily ambient temperature was significantly associated with relative humidity (*r* = −0.026, *P* < 0.001).

**Table 1:** Summary descriptive statistics of study variables in 70 study sites in Hunan, 2013‑2017

Type	Mean	SD	Min	*P*25	*P*50	*P*75	Max
Total CVD	10.1	7.5	0	5.0	8.8	13.7	225.5
Sex							
Male	11.4	10.2	0	4.2	9.4	16.1	255.2
Female	8.8	8.5	0	2.7	6.9	12.6	194.2
Age (years)							
0‑64	4.1	5.3	0	0	2.7	6.5	122.8
≥ 65	63.6	48.9	0	31.1	55.0	85.6	1760.6
Cause-specific							
HP	2.9	3.6	0	0	1.9	4.4	100.2
IHD	1.2	2.4	0	0	0	1.7	94.8
CED	3.7	4.2	0	0	2.6	5.4	113.4
HS	1.6	2.8	0	0	0	2.4	56.5
IS	0.8	1.7	0	0	0	1.1	100.2
Meteorological variable							
Daily mean temperature (°C)	17.7	8.3	−3.5	10.4	18.5	24.7	34.5
Maximum temperature (°C)	22.4	9.2	−8.2	14.8	23.4	30.1	41.9
Minimum temperature (°C)	15.0	8.1	−12.7	8.1	15.6	22.1	32.5
Relative humidity (%)	77.8	11.0	30.7	70.5	78.9	86.1	100
PM_10_ (μg/m^3^)	85.5	44.7	8.3	54.3	75.4	107.8	530.6

*CVD* total cardiovascular disease, *HP* hypertension, *IHD* ischemic heart disease, *CED* cerebrovascular disease, *HS* hemorrhagic stroke, *IS* ischemic stroke

### Exposure-response associations of ambient temperatures with YLL rates

**Fig. 2:**
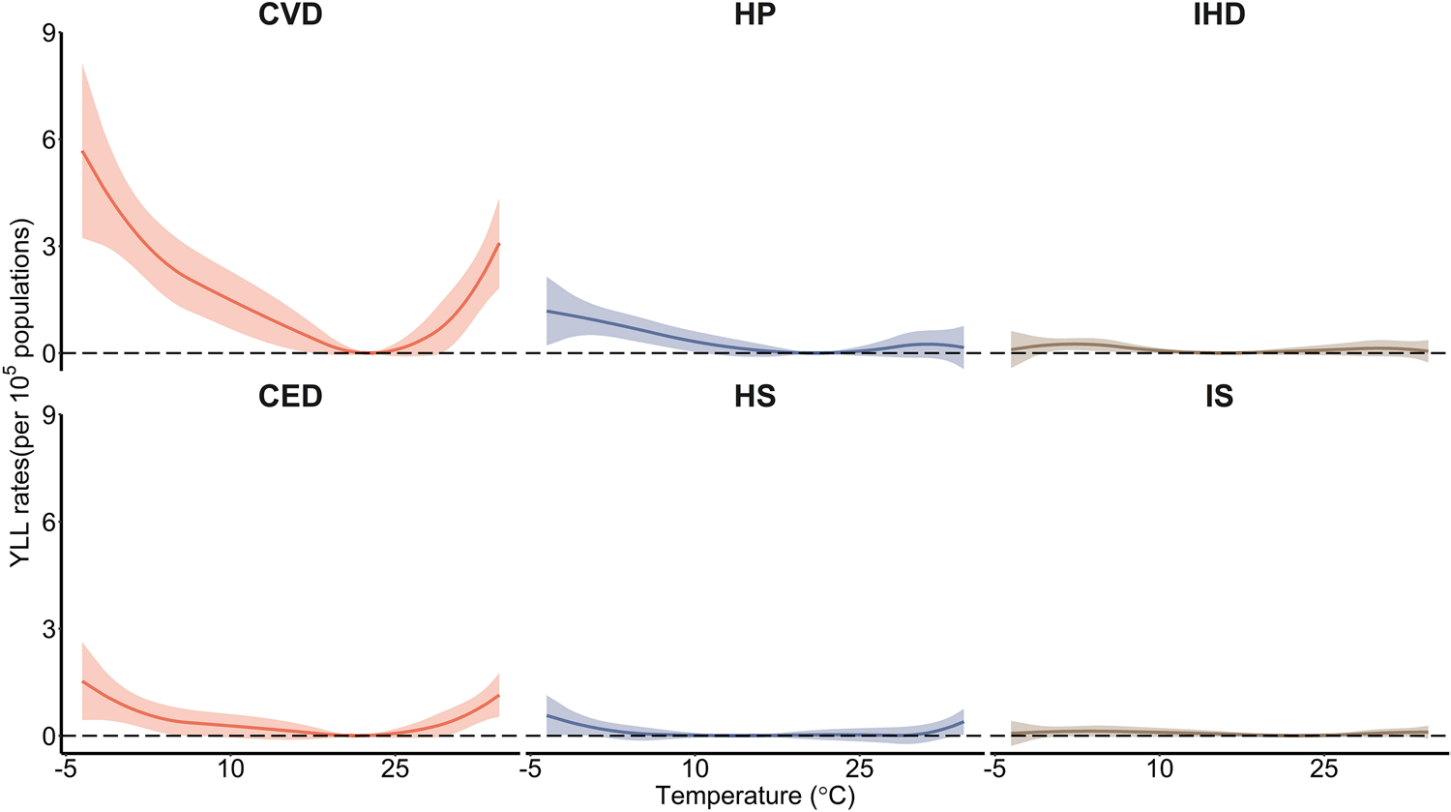
The pooled cumulative exposure-response curves between ambient temperature and YLL rate of CVD and cause-specific CVDs. Solid line, mean YLL rate of mortality. Shaded area, 95% confidence interval. CVD, total cardiovascular disease; HP, hypertension; IHD, ischemic heart disease; CED, cerebrovascular disease; HS, hemorrhagic stroke; IS, ischemic stroke

The relationship between temperature and YLL rates of CVD and cause-specific CVDs had an inverted J or U shape, which means that both cold and heat temperatures could increase the YLL rates (Fig. 2). The strongest effects of extreme heat temperature for cause-specific CVDs appeared in CED, followed by HP.

**Fig. 3:**
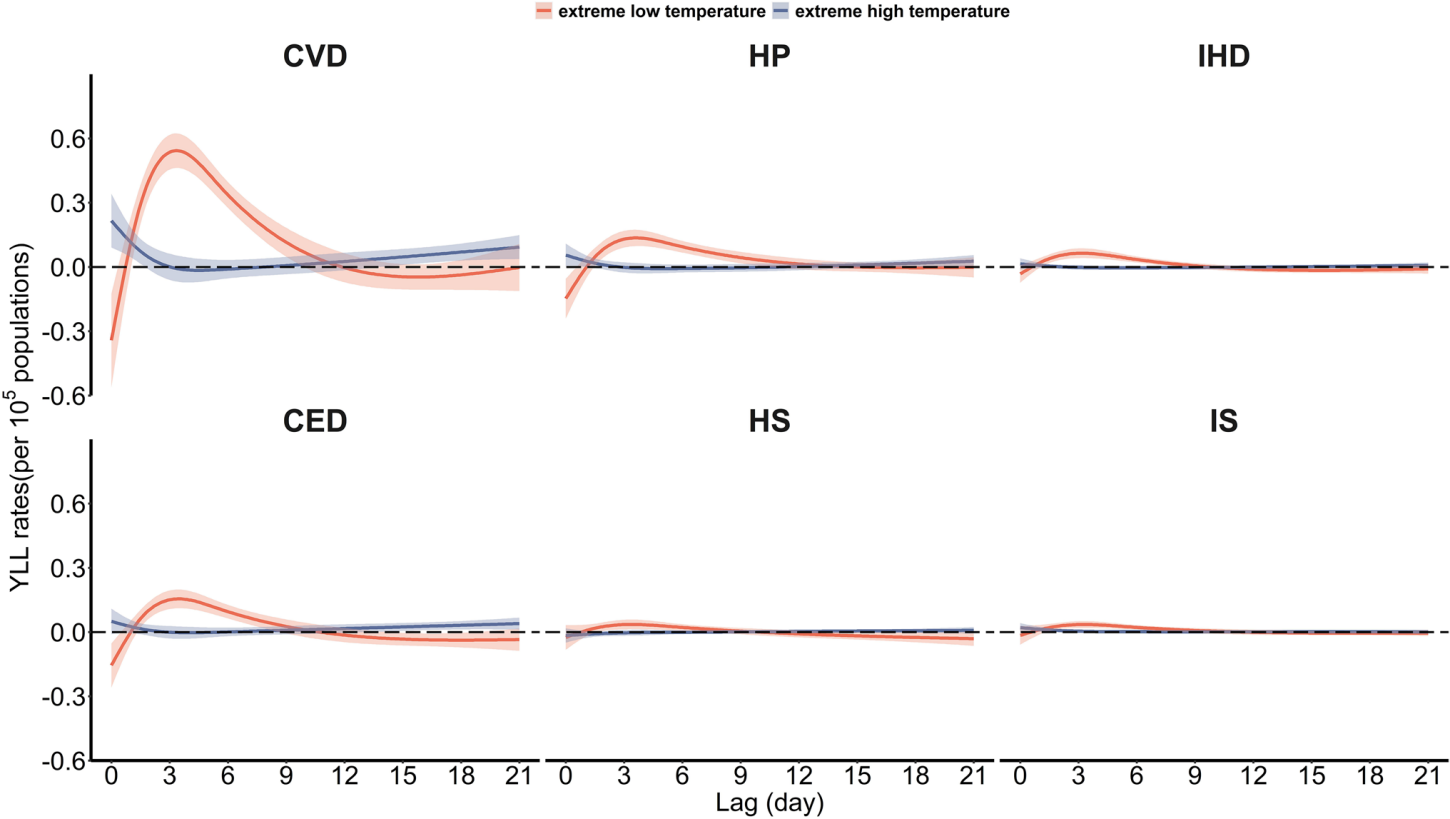
Overall lag structure in effects of extreme ambient temperature on YLL rate due to CVD and cause-specific CVDs. Solid line, mean YLL rate of mortality. Shaded area, 95% confidence interval. CVD, total cardiovascular disease; HP, hypertension; IHD, ischemic heart disease; CED, cerebrovascular disease; HS, hemorrhagic stroke; IS, ischemic stroke

We also explored the delayed effects of extreme ambient temperature on YLL rates. Extreme low temperatures had much larger temperature-attributable YLL rates than extreme high temperatures. The greatest effect of extreme heat (at 31.4 °C, the 97.5th centile) generally occurred on the first day of exposure and returned to baseline levels within 3 days. However, the risk of extreme cold temperature (at 4.9 °C, the 2.5th centile) reached a peak 3 days after exposure and declined slightly with a delayed effect even after 10 to 12 days (Fig. 3).

### Life loss per death attributable to non-optimum ambient temperature

An average of 1.89 (95% CI, 1.21‑2.56) life loss per CVD death was associated with non-optimum ambient temperature, out of which 1.13 (95% CI, 0.89‑1.37) years were attributable to cold, particularly moderate cold (1.00 years, 95% CI, 0.78‑1.23). The temperature-attributable life loss per CVD death for males (2.07 years, 95% CI, 1.44‑2.68) and the younger population (3.72 years, 95% CI, 2.06‑5.46) was relatively higher than that for females (1.88 years, 95% CI, 1.21‑2.57) and the elderly population (1.69 years, 95% CI, 1.28‑2.10) (Table 2). We also explore the effect modification of effect estimates for different subgroups. For total ambient temperature, younger population were more vulnerable than elderly population, gender were found to have weak modification effects on the YLL per death (Supplementary Table 1). For cause-specific CVDs, the greatest life loss caused by non-optimum temperature was observed for IS (2.96 years, 95% CI, 1.50‑4.33), followed by HP (2.53 years, 95% CI, 1.79‑3.25) (Table 2).

**Table 2:** Life loss per death (years, 95% CI) attributable to non-optimum ambient temperatures in 70 study sites in Hunan, 2013‑2017

Type	Total	Cold	Heat	Extreme cold	Moderate cold	Moderate heat	Extreme heat
Total CVD	1.89 (1.21, 2.56)	1.13 (0.89, 1.37)	0.76 (0.13, 1.37)	0.12 (0.11, 0.14)	1.00 (0.78, 1.23)	0.67 (0.06, 1.27)	0.09 (0.07, 0.11)
Sex							
Male	2.07 (1.44, 2.68)	1.45 (1.11, 1.75)	0.62 (0.06, 1.16)	0.13 (0.12, 0.15)	1.31 (0.99, 1.61)	0.54 (-0.01, 1.06)	0.08 (0.06, 0.10)
Female	1.88 (1.21, 2.57)	1.03 (0.82, 1.27)	0.85 (0.19, 1.55)	0.12 (0.10, 0.14)	0.91 (0.70, 1.12)	0.75 (0.11, 1.43)	0.10 (0.08, 0.12)
Age of death (years)							
0‑64	3.72 (2.06, 5.46)	2.40 (1.03, 3.66)	1.33 (0.20, 2.50)	0.25 (0.22, 0.29)	2.14 (0.81, 3.37)	1.21 (0.12, 2.32)	0.11 (0.06, 0.17)
≥ 65	1.69 (1.28, 2.10)	1.18 (0.98, 1.36)	0.51 (0.14, 0.89)	0.11 (0.10, 0.12)	1.06 (0.88, 1.24)	0.43 (0.07, 0.80)	0.08 (0.07, 0.09)
Cause-specific							
HP	2.53 (1.79, 3.25)	1.89 (1.26, 2.50)	0.64 (0.24, 1.03)	0.14 (0.12, 0.17)	1.74 (1.13, 2.35)	0.59 (0.19, 0.96)	0.06 (0.04, 0.08)
IHD	1.44 (0.70, 2.16)	0.46 (0.23, 0.70)	0.98 (0.32, 1.65)	0.03 (0.02, 0.03)	0.44 (0.21, 0.67)	0.94 (0.30, 1.60)	0.03 (0.01, 0.05)
CED	1.36 (0.75, 1.93)	0.68 (0.41, 0.95)	0.69 (0.12, 1.20)	0.09 (0.07, 0.10)	0.59 (0.33, 0.85)	0.59 (0.04, 1.09)	0.10 (0.07, 0.12)
HS	1.25 (0.60, 1.92)	0.52 (0.14, 0.92)	0.73 (0.17, 1.25)	0.08 (0.05, 0.11)	0.44 (0.09, 0.82)	0.65 (0.12, 1.15)	0.08 (0.06, 0.10)
IS	2.96 (1.50, 4.33)	0.13 (−0.03, 0.31)	2.82 (1.38, 4.18)	0.03 (0.02, 0.04)	0.10 (−0.05, 0.27)	2.73 (1.33, 4.06)	0.09 (0.06, 0.13)

*CVD* total cardiovascular disease, *HP* hypertension, *IHD* ischemic heart disease, *CED* cerebrovascular disease, *HS* hemorrhagic stroke, *IS* ischemic stroke

### Sensitivity analysis

When we changed the *dfs* of long-term trends, the maximum lag day and adjusted the model with or without PM_10_, the associations between ambient temperature and YLL rates were generally robust (Supplementary figure 1).

## Discussion

In this study, we used the YLL rate to quantify the association between temperature and mortality of CVDs. Nonlinear associations of the temperature-YLL rate relation were found in an inverted J or U shape, with an increased YLL rate for both high and low temperatures, which is consistent with previous studies that used relative risk or attributable fractions as health outcomes (Fu et al. 2018; Huber et al. 2020; Ma et al. 2020; Moghadamnia et al. 2018).

Our study explored the mean YLL per death of CVDs caused by temperature, and a mean of 1.89 YLL per death of CVDs was attributable to temperatures in the selected counties within the study period. Our dataset including five different cause-specific CVD deaths enables us to find variations in cause-specific temperature effects. We quantified the average life loss per death of cause-specific CVDs, and found that temperature exposure caused a higher mean life loss for patients with IS than HP, with means of 2.96 and 2.53, respectively. We also analyzed YLL rates caused by different temperature components. The findings showed that the majority of life loss of CVDs (1.13 years) occurred on cold days below the minimum YLL temperature, and most were from moderate cold. There could be several reasons for the large fraction of moderate cold effects: (1) prolonged lag effects and the high frequency of moderate cold temperatures; (2) people living in subtropical climate regions, such as Hunan province, central China, may have a higher sensitivity to cold weather and adaptability to hot temperatures (Liu et al. 2020); and (3) exposure to cold temperatures can lead to increased blood pressure, blood viscosity, cardiac load, and serum low-density lipoprotein-cholesterol concentration, all of which contribute to cardiovascular mortality (Gostimirovic et al. 2020; Hong et al. 2012; Lim et al. 2015; Yang et al. 2015). Therefore, appropriate strategies should be established to reduce the mortality burden from the whole range of ambient temperatures.

Recently, climate change has been one of the greatest global health threats (Watts et al. 2017). Extreme temperature events become increasingly frequent. Many studies have provided evidence that extreme temperatures are associated with short-term increases in daily mortality (Adab et al. 2021; Chen et al. 2014; Curtis et al. 2017). Our study adds evidence that extreme temperatures also have an impact on YLL. A mean of 0.12 and 0.09 YLL per CVD patient was attributable to extreme cold and heat temperatures. Our study also showed that extreme heat effects on YLL of CVD occur immediately, while extreme cold effects last longer, which is consistent with the analyses of temperature–mortality studies (Chung et al. 2015; Onozuka and Hagihara 2015). Generally, understanding the lag pattern between temperature exposure and death is important to develop adaptive responses for extreme events.

Moreover, we explored potential effect modification in terms of demographic characteristics, such as sex and age. We found a greater YLL per CVD death in males than in females, indicating that men were more susceptible to non-optimum ambient temperatures than women. Nevertheless, a systematic review showed that women were more sensitive to the effects of ambient temperature on cardiovascular mortality and morbidity than men (Moghadamnia et al. 2017). A previous study also reported that the heat-attributable fraction of cardiovascular deaths was higher for women and the cold-attributable fraction was larger in men (Achebak et al. 2019). The different physiological and behavioral factors in different regions could explain the discrepancy among the studies. In addition, men are more likely to be exposed to hazardous substances, which results in higher YLL values for males. We also found a higher temperature-related life loss per CVD death in the younger population than in the elderly population during the study period. A low percentage of young people may suffer from CVDs. However, YLL can give more weight to deaths among younger patients; therefore, the mortality burden of each death is heavier (Hu et al. 2021). These findings suggest that when we use YLL per death as a measure, we should pay more attention to men and young people in planning adaptation policies, public health interventions and social services to alleviate the impact of temperature.

Some limitations of our study deserve mention. First, the data we used were limited to Hunan province, central China, and did not include more regions; therefore, the findings may lack generalizability. However, we applied population-related YLL rates as health outcomes; therefore, the impacts of ambient temperature on YLL rates in different regions were comparable. Second, similar to many previous studies, some errors cannot be avoided. The study was essentially an ecological study, which could not control individual-level confounders and might have resulted in ecological bias. We used temperature data from weather monitoring stations rather than individual direct measurements, which could lead to exposure measurement bias.

## Conclusions

In summary, we evaluated the corresponding life loss of CVDs attributable to non-optimum temperatures. We found that cold temperature had a stronger impact than heat and showed that moderate cold temperature represented most of the life loss. Our study highlights that public health interventions for climate change are necessary.

## Supplementary information

ESM 1 (DOCX 14 kb)

ESM 2 (JPEG 2623 kb)

## Data Availability

The meteorological data can be obtained from the China Meteorological Data Sharing Service System (http://data.cma.cn/). The mortality data are not publicly available due to the information that could compromise the personal privacy, but the data that support the findings of this study are available from the corresponding author (hncdc_zcl@163.com) upon reasonable request.

## Abbreviations

CVD: Cardiovascular disease
YLL: Year of life lost
MYT: Minimum YLL rates temperature
HP: Hypertension
IHD: Ischemic heart disease
CED: Cerebrovascular disease
HS: Hemorrhagic stroke
IS: Ischemic stroke
df: Degree of freedom
