# Supplementary material for: Impact of ambient temperature on life loss per death from cardiovascular diseases: a multicenter study in central China
Source: Environ Sci Pollut Res Int. 2021 Oct 11;29(11):15791–9. doi: 10.1007/s11356-021-16888-7 (PMC8827384; doi:10.1007/s11356-021-16888-7)
Supplement: Supplementary file 1 — (DOCX 14 kb) [file 11356_2021_16888_MOESM1_ESM.docx]

**Supplementary table 1.** The relative life loss per death for effect estimates of ambient temperature among two different subgroups.

| Type | Total | Cold | Heat | Extreme cold | Moderate cold | Moderate heat | Extreme heat |
| --- | --- | --- | --- | --- | --- | --- | --- |
| Sex |  |  |  |  |  |  |  |
| Male | 1.10(0.68-1.79) | 1.41(1.03-1.93)* | 0.73(0.12-4.48) | 1.08(0.86-1.33) | 1.44(1.03-2.02)* | - | 0.80(0.58-1.11) |
| Female | 1 | 1 | 1 | 1 | 1 | 1 | 1 |
| Age of death (years) |  |  |  |  |  |  |  |
| 0-64 | 2.20(1.27-3.80)* | 2.03(1.06-3.91)* | 2.61(0.54-12.47) | 2.27(1.93-2.68)* | 2.02(0.97-4.20) | 2.81(0.41-19.15) | 1.38(0.80-2.35) |
| ≥65 | 1 | 1 | 1 | 1 | 1 | 1 | 1 |

Note: asterisk (*) denotes differences between different subgroups were significant at α=0.05.
